# Pentameric quaternary structure of the intracellular domain of serotonin type 3A receptors

**DOI:** 10.1038/srep23921

**Published:** 2016-04-05

**Authors:** Akash Pandhare, Petar N. Grozdanov, Michaela Jansen

**Affiliations:** 1 Department of Cell Physiology and Molecular Biophysics and Center for Membrane Protein Research, Texas Tech University Health Sciences Center, Lubbock, Texas 79430, USA; 2 Department of Cell Biology and Biochemistry, Texas Tech University Health Sciences Center, Lubbock, Texas 79430, USA

## Abstract

In spite of extensive efforts over decades an experimentally-derived structure of full-length eukaryotic pentameric ligand-gated ion channels (pLGICs) is still lacking. These pharmaceutically highly-relevant channels contain structurally well-conserved and characterized extracellular and transmembrane domains. The intracellular domain (ICD), however, has been orphaned in structural studies based on the consensus assumption of being largely disordered. In the present study, we demonstrate for the first time that the serotonin type 3A (5-HT_3A_) ICD assembles into stable pentamers in solution in the absence of the other two domains, thought to be the drivers for oligomerization. Additionally, the soluble 5-HT_3A_-ICD construct interacted with the protein RIC-3 (resistance to inhibitors of cholinesterase). The interaction provides evidence that the 5-HT_3A_-ICD is not only required but also sufficient for interaction with RIC-3. Our results suggest the ICD constitutes an oligomerization domain. This novel role significantly adds to its known contributions in receptor trafficking, targeting, and functional fine-tuning. The innate diversity of the ICDs with sizes ranging from 50 to 280 amino acids indicates new methodologies need to be developed to determine the structures of these domains. The use of soluble ICD proteins that we report in the present study constitutes a useful approach to address this gap.

Recent X-ray and cryo-electron microscopy structures of glutamate-gated chloride channel (GluClα)^1^, γ-aminobutyric acid (GABA_A_β3)^2^, nicotinic acetylcholine (nAChR)^3^, glycine (Glyα1)^4^, and serotonin type 3A (5-HT_3A_)^5^ receptors have demonstrated high structural conservation of the extracellular (ECD) and transmembrane domains (TMD) of pLGICs. However, in these structures the intracellular domain (ICD) was either completely or partially removed. The 5-HT_3A_ structure was obtained after proteolytic removal of a 61 amino-acid-long loop preceding the conductance-limiting MA-helix resulting in a 40-fold increased single-channel conductance compared to wildtype channels. We observed a similar increase previously in a construct in which the entire ICD was replaced by a heptapeptide^6^. At present a complete structural understanding of the ICD of pLGICs is missing. Importantly, the great diversity in length and amino acid composition of the ICD indicates, that each of the more than 40 pLGIC subunits found in humans may have unique structural and functional features. Here we investigate a soluble 5-HT_3A_ intracellular domain construct, 5-HT_3A_-ICD, that neither contains ECD nor TMD. 5-HT_3A_-ICD can be expressed in *E. coli* and purified to homogeneity in quantities amenable for detailed structural studies. Surprisingly, we demonstrate stable pentameric assembly in solution of 5-HT_3A_-ICD indicating that the ICD alone may be not only a driver for oligomerization but also a candidate for mediating oligomerization of specific subunits in specific stoichiometries.

## Results and Discussion

**Figure 1:**
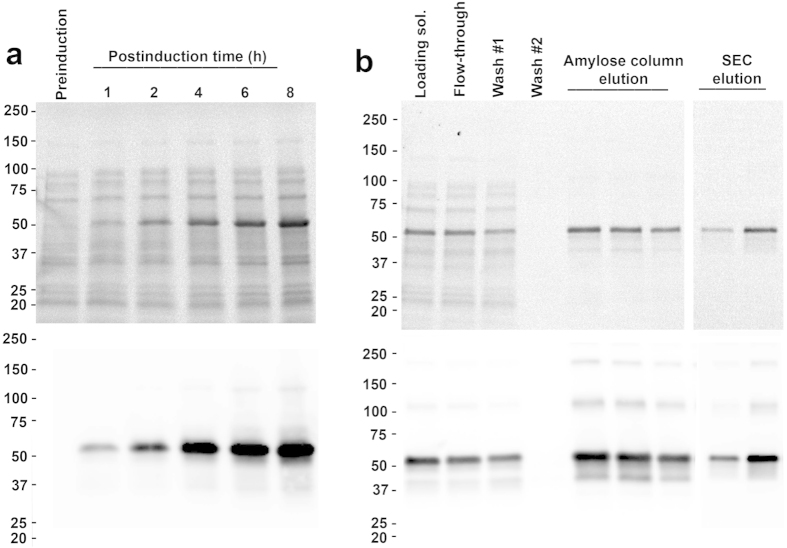
Optimization of expression and purification of 5-HT_3A_-ICD. SDS-PAGE gels of protein expression samples, loading normalized against respective cell densities using OD600. Stain-free SDS-TGX-gel, top, and Western blot, bottom. (**a**) Optimization of induction time. Samples were processed from un-induced (preinduction) cultures and after induction of indicated duration. (**b**) Amylose column purification and size exclusion chromatography (SEC) samples.

The 5-HT_3A_-ICD, a segment of 115 amino acids as determined by multiple sequence alignment with all pLGIC sequences and defined as the sequence between the third (TM3) and fourth transmembrane segments (TM4), was fused to the C-terminus of a modified maltose binding protein (MBP)^7^. To reduce the length of a potentially flexible linker, MBP was terminated after Q367 in the C-terminal α-helix, and the linker between MBP and 5-HT_3A_-ICD consisted of a short alanine linker. The 5-HT_3A_-ICD sequence starts after the α-helical TM3 with a loop and ends with an α-helical structure, the membrane associated (MA) helix, also called amphipathic helix (HA), that is preceding and continuous with the fourth and last α-helical transmembrane segment (TM4) of the transmembrane domain of full-length receptors^5^. The chimeric 5-HT_3A_-ICD construct, hereafter referred to as 5-HT_3A_-ICD, was expressed in *E. coli* and purified from the soluble fraction after cell disruption by affinity chromatography using amylose resin and size exclusion chromatography (SEC) (Fig. 1). The presence of the complete ICD was confirmed by mass spectrometry (MS/MS, MALDI-TOF/TOF 4800 mass spectrometer, Applied Biosystems). On SDS-PAGE gels the protein ran at 53 kDa, while higher molecular weight bands approximately every 50 kDa were discernible clearly with Western blot detection.

**Figure 2:**
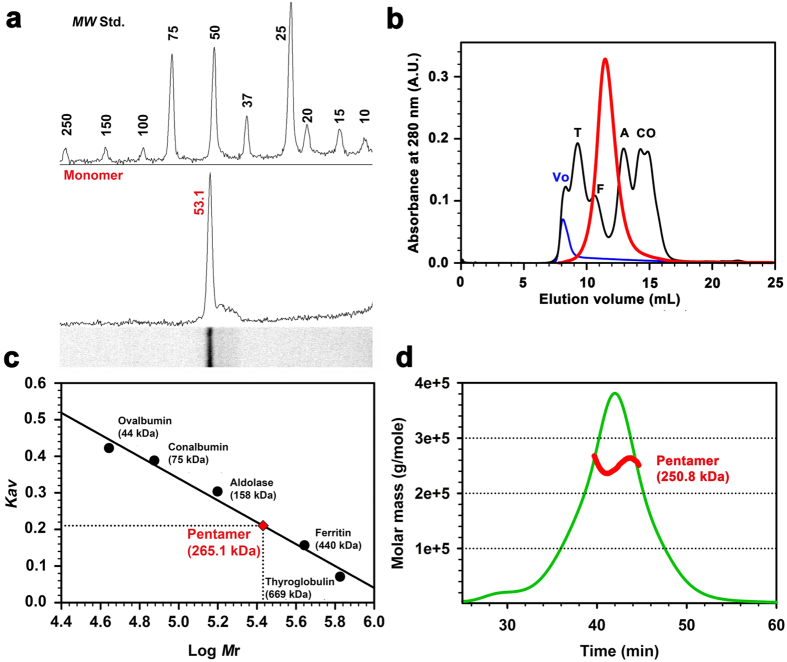
5-HT_3A_-ICD forms pentameric assemblies in solution. (**a**) The weight of the 5-HT_3A_-ICD monomer construct was determined to be 53 kDa using SDS-gel electrophoresis. Molecular weight standards, top, 5-HT_3A_-ICD, bottom. (**b**,**c**) Size-exclusion chromatography (SEC) yielded a weight of 265.1 kDa for 5-HT_3A_-ICD. Chromatogram of standards, black, blue dextran, blue, and 5-HT_3_ _A_-ICD sample, red. (**d**) SEC-MALS confirmed pentameric assembly with the weight-average molar mass of 5HT_3A_-ICD as 250.8 ± 4.4 kDa. A representative SEC-MALS profile showing the Rayleigh ratio (green line) and the weight-average molar mass (red line) computed from light scattering.

**Figure 3:**
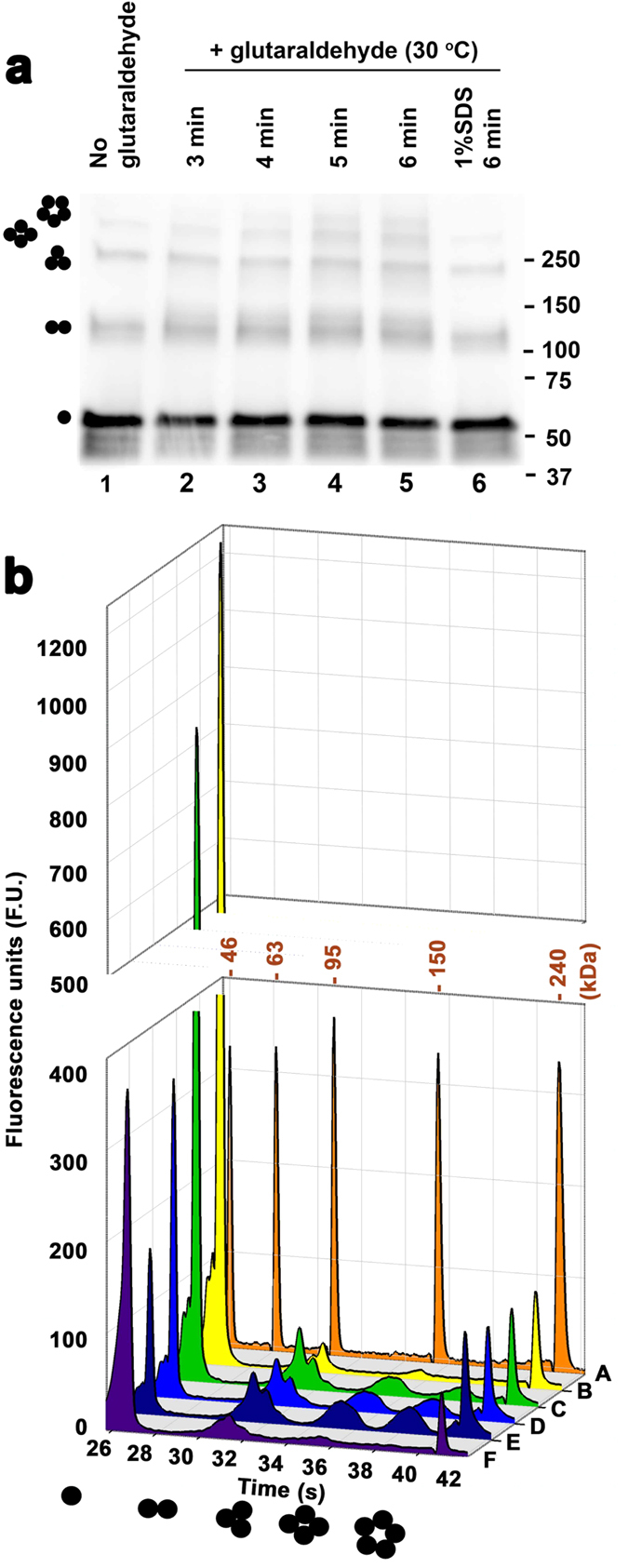
Glutaraldehyde cross-linking indicates pentameric assembly of 5-HT_3A_-ICD. Samples were cross-linked with glutaraldehyde using a vapor diffusion method. Oligomeric states corresponding to the observed signals are indicated by monomer to pentamer cartoons. (**a**) Samples were treated for the durations indicated at 30 °C. The SDS sample, 6, was pretreated with 1% SDS before crosslinking. Samples were separated by SDS-PAGE and detected with Western Blot. (**b**) Samples were cross-linked for 0, 3, 4, or 5 minutes at 30 °C (B–E), or pretreated with 1% SDS before crosslinking for 10 minutes. Chromatogram A contains molecular weight markers as indicated for each peak, and B–F contain markers for 46 and 240 kDa, only. Samples were separated by capillary gel electrophoresis.

Using SDS-PAGE and denaturing microfluidic capillary gel electrophoresis (Bioanalyzer, Agilent) the weight of purified monomeric 5-HT_3A_-ICD construct was determined to be 53 kDa (theoretical weight 54 kDa) (Figs 2 and 3). The peak fraction analyzed by SEC eluted as a single symmetric peak indicating assembly into a defined and stable oligomeric state. The elution volume (11.52 ± 0.02 mL, n = 5) of 5-HT_3A_-ICD was significantly smaller than what would correspond to monomeric 5-HT_3A_-ICD, indicating higher oligomeric assemblies. MBP elutes at 15.2 mL yielding a predicted weight of 44 kDa (theoretical weight 41 kDa), indicating that MBP is a monomeric protein under the experimental conditions tested^7^,^8^. Importantly, MBP has been extensively used to create fusion proteins for structural studies, including peptides of similar or even shorter lengths as studied here (Tom20, 61 amino acids^9^; MCL1, 147 amino acids^10^; PilA1, 133 amino acids^11^; Norrin, 103 amino acids^12^, Menangle virus phosphoprotein, 49 amino acids^13^; adenylate cyclase 1 Receptor-short N-terminal extracellular domain, 93 amino acids^14^). If oligomeric assemblies were observed in X-ray structures of MBP fusion proteins, oligomerization was always mediated by the fusion protein of interest and not MBP. The elution volume of 5-HT_3A_-ICD chimera during SEC corresponds to a weight of 265.1 ± 2.8 kDa (n = 5), indicative of a pentameric assembly. When the purified protein was treated with the cross-linker glutaraldehyde using a vapor diffusion method^15^ and then separated by either SDS-PAGE or denaturing capillary gel electrophoresis, a ladder was observed, representing monomer, dimer, trimer, tetramer and pentamer (Fig. 3). Pretreatment of 5-HT_3A_-ICD with SDS obliterated the higher oligomeric assemblies (Fig. 3). Dynamic light scattering (DLS) experiments yielded a particle size corresponding to 245 ± 19 kDa (n = 5). To further ascertain the results obtained by SEC, chemical cross-linking and DLS, we performed SEC coupled with multi-angle light scattering (SEC-MALS), a molecular shape-independent method, to directly establish the absolute molecular weight of purified protein in solution. Analysis of four separate experiments yielded a weight-average molecular mass of 250.8 ± 4.4 kDa (mean ± S.E.M., n = 4) with a dispersity term of 1.010 ± 0.004 (mean ± S.E.M., n = 4) (Fig. 2, Table 1), again confirming the pentameric assembly of 5HT_3A_-ICD. The result that the 5-HT_3A_-ICD chimera conferred pentameric assembly is surprising.

In pentameric ligand-gated ion channels (pLGICs), the three domains found along an axis perpendicular to the membrane are the extracellular (ECD), transmembrane (TMD), and intracellular domain (ICD). Structurally, at the level of each domain within a receptor, five-fold symmetry is found. Previously, ECDs and TMDs have been expressed individually and their oligomerization studied. The present study is the first to show pentameric assembly of the ICD alone. Originally, intersubunit-interactions between ECDs were thought to drive the assembly and stoichiometry of pLGICs^16^,^17^,^18^,^19^. This suggested that the ECD would be an autonomous folding and assembly domain. Indeed, AcetylCholine Binding Protein (AChBP)^20^, a soluble protein found in the snail *Lymnaea stagnalis*, that is homologous to the ECD of nAChR, forms pentameric assemblies in solution. Similarly, engineered ECD-only constructs of pLGICs that natively form functional homopentamers, including GlyRα1 and nAChRα7 subunits, assemble into pentamers in solution, that preserve high-affinity ligand-binding capability^21^,^22^. Conversely, and unsurprisingly, the ECD of nAChRα1 that normally is found in heteropentamers does not assemble to higher homooligomeric states and in the X-ray structure nAChRα1 ECD was monomeric^23^. In summary, these individual ECDs parallel the oligomerization properties of their full-length receptors.

Conversely, a construct of the prokaryotic *Gloeobacter violaceus* pLGIC (GLIC) that solely consists out of the ECD is monomeric in solution even at very high concentration and only forms hexa- but not pentameric structures in the crystallized form^24^. Full-length GLIC that consists of ECD and TMD has so far been only described as a pentamer^25^,^26^. It was postulated that the GLIC TMD is required for correct pentameric assembly^24^. Indeed, for diverse eukaryotic pLGICs, aromatic residues in non-channel-lining transmembrane helices (TM1, TM3, and TM4) network to determine α-helical packing and thus subunit stoichiometry^27^. Several pLGIC TMDs expressed individually assemble as pentamers in lipid micelles. For example, cation-conducting nAChRα7 TMDs as well as nAChRα4β2 TMDs with short M3-M4 linkers, GGGEG and G_5_, respectively, assemble as pentamers in lauryldimethylamine-oxide (LDAO) micelles^28^,^29^, and anion-conducting GlyR TMDs form pentamers in lysophospholipid lyso-1-palmitoylphosphotidylglycerol (LPPG)^30^. Hydrophobic contacts, especially leucine-isoleucine, and leucine-leucine interactions, drive these assemblies. Interestingly, separate nAChR α4 and β2 TMDs alone also form homopentamers, whereas the complete subunits do not form such homopentamers, and instead only obligate heteropentamers, indicating that domains apart from the TMD control the defined stoichiometric assembly in full-length receptors.

Only a few previous studies investigated expression of solely the ICD. No pentamers were observed and spectroscopic methods as well as limited digests lead to the conclusion that the ICD was unfolded^31^,^32^. In general, the ICD has largely been thought of as a region with disorganized structure^3^,^33^. Intriguingly, we show here, that the complete 5-HT_3A_-ICD when fused to the soluble monomeric MBP confers pentameric architecture to the resulting soluble chimera. Only stable and defined pentamers were observed, without evidence for other oligomeric assembly states. The driving force behind this observed pentamerization must lie in sequences directly contributed by the ICD.

**Figure 4:**
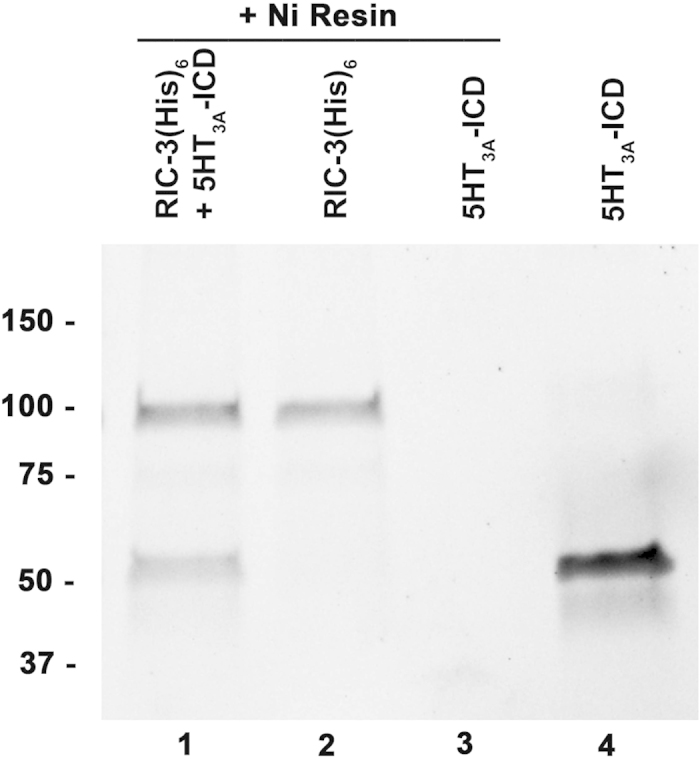
5-HT_3A_-ICD interacts with chaperone protein RIC-3. RIC-3 with a C-terminal (His)_6_ (hRIC-3(His)_6_) pulled down 5-HT_3A_-ICD using nickel metal-affinity resin.

RIC-3 is a crucial chaperone protein for some pLGICs that influences their functional maturation^34^. For mouse 5-HT_3A_ receptors expressed in *Xenopus laevis* oocytes we have shown that RIC-3 co-expression attenuated serotonin-induced currents almost completely, as compared to 5-HT_3A_ expressed alone. When the 115-amino acid 5-HT_3A_-ICD is replaced with the short heptapeptide M3-M4 linker of GLIC, this RIC-3 effect is abolished^6^. In reverse chimeras consisting of GLIC-ECD and TMD with the 5-HT_3A_-ICD added to obtain a GLIC-5-HT_3A_-ICD chimeras we showed that RIC-3 co-expression similarly only modulated the 5-HT_3A_-ICD-containing chimeras, but not GLIC^35^,^36^. These results indicate that the 5-HT_3A_-ICD is required for RIC-3 modulation. By using both proteins, GLIC-5-HT_3A_-ICD and RIC-3, individually purified after expression in *E. coli,* in pull-down experiments, we showed that GLIC-5-HT_3A_-ICD interacts with RIC-3^37^. Under the same conditions wild-type GLIC that does not contain any ICD is not pulled down. These experiments demonstrate that the interaction between the 5-HT_3A_-ICD and RIC-3 is direct and not mediated by other proteins. Here, the soluble 5-HT_3A_-ICD chimera was used in pull-down experiments using metal affinity resin directed towards a C-terminal His_6_ tag in a RIC-3 construct (hRIC-3(His)_6_). The soluble 5-HT_3A_-ICD chimera was successfully pulled down in these experiments providing evidence that the 5-HT_3A_-ICD is not only required but also sufficient for the interaction with RIC-3 (Fig. 4). These studies can be extended to the multitude of other intracellular proteins that interact with pLGIC ICDs. The characterization of such interaction sites may provide targets for future drug design.

It is astounding that the ICD that is still thought to be a vastly disordered domain drives pentamerization when expressed alone, and it is intriguing to propose that it is also a driver for pentamerization of pLGICs as a whole. Importantly, quaternary polymorphism as observed for many other oligomeric proteins including the GLIC ECD is absent for the 5-HT_3A_-ICD. The soluble 5-HT_3A_-ICD chimera construct is now a perfect starting point for determining its high-resolution structure and also for identification of molecular features inside the ICD that mediate RIC-3 as well as other cytosolic protein interactions.

The recent 5-HT_3A_ X-ray structure including the proteolyzed ICD showed that lateral windows identified as ion pathways in the *Torpedo* nAChR model connecting the intracellular channel cavity with the cytosol were entirely occluded by post-M3 loops^5^,^38^. Additionally, three MA-helix arginines previously thought to electrostatically repel conducted cations and be therefore causative for the sub-pSiemens single-channel conductance were involved in inter-subunit salt bridges^5^,^38^. The structure does also not provide an intracellular pathway for cations, albeit the crystallized construct had a significantly increased single-channel conductance^5^. Clearly, further detailed studies of the 5-HT_3A_-ICD are therefore needed to elucidate the intracellular ion pathway as well as opening mechanism, and to reconcile previous functional studies with structure. Due to the diversity of the ICD in this large ion-channel superfamily, more than 40 subunits in humans, investigations are needed for diverse pLGICs. Highly-specialized mechanisms for the fine-tuning of targeting, trafficking or even functional aspects have evolved to be increasingly complex in metazoans over unicellular organisms. Importantly, it is becoming increasingly apparent that the ICD is involved in all aspects of pLGIC from folding and structural assembly to modulation of agonist efficacy and desensitization.

## Methods

### Plasmids

The cytoplasmic domain of the mouse 5-HT_3A_ receptor fused to the C-terminus of maltose binding protein (MBP) was generated using a modified pMAl-c2x (New England Biolabs) vector, pMALX^7^. The 5-HT_3A_-ICD (QDL…VGY) was amplified by PCR using appropriate NotI and BamHI forward and reverse primers, respectively. The BamHI reverse primer also included a stop codon. The final construct, MBP-5-HT_3A_-ICD-pMALX, was obtained after double-digest of the PCR-amplified ICD as well as pMALX, and subsequent ligation.

### Protein Expression and purification

The MBP-5-HT_3A_-ICD fusion protein was overexpressed in *Escherichia coli* (*E. coli*), BL21-CodonPlus^®^ (DE3)-RIPL cells (Agilent Technologies; Santa Clara, CA). For large scale purification cells were harvested by centrifugation (4,600 × *g* for 15 min at 4 °C) and resuspended in lysis buffer (Buffer A; 20 mM Tris, 200 mM NaCl, 1 mM ethylenediaminetetraacetic acid (EDTA), pH 7.4) containing a freshly prepared cocktail of protease inhibitors (1 mM phenylmethylsulfonyl fluoride (PMSF); Research Products International Corp., 10 μg/mL leupeptin; Sigma-Aldrich, 7 μg/mL pepstatin; Fisher Scientific), and lysozyme (1 mg/mL; Sigma Aldrich) and DNaseI (20 μg/mL; Sigma-Aldrich). The cell suspension was passed through an EmulsiFlex-C3 high-pressure homogenizer (AVESTIN, Inc., Ottawa, ON, Canada) to achieve cell lysis. Debris was removed by centrifugation (10,000 × *g* for 40 min at 4 °C) and the lysate was subsequently clarified by a second round of centrifugation for 1 h at 90,000 × *g* and 4 °C. The resultant supernatant was loaded onto a gravity-packed amylose-resin column (New England BioLabs, Inc.). Unbound proteins were washed extensively with buffer A, and the bound fusion protein was eluted with buffer A containing 20 mM maltose.

### Size Exclusion Chromatography

The amylose column purified fusion protein was subjected to size exclusion chromatography for additional purification and/or molecular weight determination on a Superdex^TM^ 200 10/300 GL column (GE Healthcare) equilibrated with sizing column buffer B (20 mM HEPES, 150 mM NaCl, 0.02% NaN_3_, pH 7.4). Elution of protein was monitored by UV absorbance at 280 nm (*A*_280_). The yield of highly-pure 5-HT_3A_-ICD chimera achieved was 18 mg per L of culture. For calibration of the Superdex 200 column, high molecular weight standard proteins (thyroglobulin 669 kDa, ferritin 440 kDa, aldolase 158 kDa, conalbumin 75 kDa and ovalbumin 44 kDa) and Blue Dextran 2000 were used according to the manufacturer’s instructions (GE Healthcare). Blue Dextran 2000 was used to determine the column void volume (***V***_**o**_). The value of the gel-phase distribution coefficient (***K***_**av**_) was calculated for each standard as well as the fusion protein using the equation:


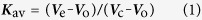


where ***V***_**e**_ is the elution volume, ***V***_**o**_ is the column void volume (8.16 mL), and ***V***_**c**_ is the geometric column volume (24 mL). For each standard protein, the calculated ***K***_**av**_ value was plotted against the log of its known molecular weight (Log *M*_r_). The standard curve analysis algorithm of SigmaPlot 13.0 (Systat Software Inc., San Jose, CA) was then employed to obtain a predicted value (***x***) of the molecular weight of the fusion protein using a linear equation:


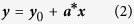


where ***a*** is the slope and ***y***_**0**_ is the y-intercept. The obtained molecular weight of the fusion protein represents the average of at least five separate experiments.

### Chemical Cross-Linking

For investigating the oligomeric state of fusion protein, glutaraldehyde-mediated cross-linking experiments were performed at 30 °C by utilizing a vapor-diffusion technique. Briefly, 40 μL of 25 % (v/v) glutaraldehyde (Sigma-Aldrich) was placed in the bottom of a microfuge tube and acidified with 1 μL of 5 N HCl. 15 μL of buffer B containing 0.5 μg of purified fusion protein was placed on the inside of the cap of the same microfuge tube. Upon carefully closing the cap, the protein solution was allowed to form a hanging drop inside the tube, and separated at ≤1 cm from glutaraldehyde. The entire ‘closed microfuge tube system’ was then incubated in a mini dry-bath at the temperature indicated. The protein solution was then carefully transferred into a separate tube to quench the reaction by adding sodium dodecyl sulfate (SDS) sample buffer (62.5 mM Tris HCl, pH 6.8, 10% (v/v) glycerol, 2% (w/v) SDS, 100 mM dithiothreitol (DTT), 0.01% (w/v) bromophenol blue). All samples were boiled and then resolved by SDS-PAGE. Cross-linked proteins were transferred to polyvinyl di-fluoride (PVDF) membrane and immunoblotted as described later. The analysis was repeated at various intervals of time after the start of the reaction to reveal a ladder of the oligomeric states.

Alternatively, in a similar experiment, protein was exposed to glutaraldehyde vapors for various time intervals as described above and the cross-linked proteins were resolved by ‘On-Chip-Electrophoresis’ under reducing conditions using the Agilent 2100 Bioanalyzer (Agilent Technologies, Inc.). Protein chip and loading samples were prepared using the manufacturer provided kit, and according to instruction manual.

### Dynamic Light Scattering

Dynamic light scattering was used for particle size determination. Purified protein was diluted to a protein concentration of 1.0 mg/mL, filtered with a 0.22 um Millipore Millex-GV filter (Millipore, Billerica, MA) right before measurements, and subjected to the light scattering measurements at 90° using a Brookhaven Instruments (Brookhaven, CT) BI-200SM device with an avalanche photodetector. The molecular mass of the protein was calculated from the measured hydrodynamic radius and a calibration curve that included seven soluble proteins (ribonuclease A, carbonic anhydrase, bovine serum albumin, conalbumin, aldolase, ovalbumin, and IgG), and three membrane proteins (KcsA, connexin 26 hemichannels, and P-glycoprotein).

### Size Exclusion Chromatography in tandem with Multi-angle Light Scattering (SEC-MALS)

SEC-MALS analysis was utilized to determine the weight-average molar mass (*M*_w_, g mole^−1^) and dispersity (*Ð, M*_w_/*M*_n_, where *M*_n_ is the number-average molar mass) of purified protein independent of its shape. Chromatographic separation of protein samples was obtained under isocratic conditions using a Superdex^TM^ 200 HR 10/300 GL column (GE Healthcare), pre-equilibrated with two column volumes of an SEC buffer (20 mM HEPES, 150 mM NaCl, 5 mM maltose, 0.01% NaN_3_, pH 7.4). The individual run was developed at an ambient temperature while a flow rate of 0.3 mL/min was maintained on an APLC system (LabAlliance, State College, PA) equipped with a solvent degasser. The chromatography system was in a tandem arrangement downstream with a miniDAWN-TREOS light scattering instrument equipped with three detectors positioned at angles 44^º^, 90° and 136° (Wyatt Technologies, Santa Barbara, CA) and Optilab T-rEX dRI detector (Wyatt Technologies, Santa Barbara, CA). Both instruments were reading at a wavelength of 658 nm. Scattered angles were normalized using bovine serum albumin (BSA) following the manufacturer’s instructions. For a typical experiment, the SEC buffer was injected for a blank run to establish stable baselines for the light scattering and differential refractive index measurements. All purified protein samples were filtered with a 0.22 μm low protein binding Millex-GV filter just before each experimental run. Subsequently, 0.1–1.0 mg of purified and filtered protein samples were loaded onto the column and their elution profiles were monitored at *A*_280_. The extinction coefficient of purified protein was calculated from the amino acid sequence by using the ExPASy-ProtParam tool. The online measurement of the Rayleigh scattering intensity was processed to determine the weight-average molar mass and dispersity of protein material contained in top one-third of the major *A*_280_ chromatographic peak. Data was acquired with the ASTRA 5.3.4 software (Wyatt Technologies, Santa Barbara, CA) and processed using Debye model as per the manufacturer’s guidelines.

### Pull-down Assay

1X protease inhibitor cocktail (Thermoscientific) was added just before use to all buffers prepared freshly for protein ‘pull down’ assay. 4 μg of purified N-terminal MBP-tagged and C-terminal histidine-tagged human RIC-3 (hRIC-3(His)_6_) was allowed to bind to 15 μL of Ni-NTA resins, pre-equilibrated with low imidazole–containing buffer C (50 mM potassium phosphate, pH 8.0, 150 mM NaCl, 1 mM MgCl_2_, 0.05% Triton X-100, 0.05% DDM, 10 mM imidazole), at 4 °C for 30 min. After initial incubation, unbound hRIC-3(His)_6_ was washed away with buffer D (20 mM HEPES, 200 mM NaCl, 1 mM MgCl_2_, 0.05% Triton X-100, 0.05% DDM, pH 7.4) with 10 mM imidazole, and the resin-bound hRIC-3(His)_6_ was then incubated in the presence or absence of purified 20 μg of MBP-5-HT_3A_-ICD for an additional 1 h at 4 °C. Simultaneously, in another condition, 20 μg of MBP-5-HT_3A_-ICD was incubated with 15 μL of Ni-NTA resins, pre-equilibrated with buffer D for 1 h at 4 °C. After 1 h of incubation, resins were washed ten times with 300 μL of washing buffer D with 15 mM imidazole. The bound proteins were eluted with buffer D containing 250 mM imidazole, and were immediately examined by SDS polyacrylamide gel electrophoresis and Western blotting.

### SDS-PAGE and Western Blot Analysis

For SDS-electrophoresis, 4–15% precast gradient TGX Stain-Free^TM^ gels (Bio-Rad Laboratories, Inc.) were used and visualized by stain-free enabled imager (Gel Doc^TM^ EZ Imager, Bio-Rad). After blotting onto PVDF membrane (Bio-Rad), the proteins were visualized by probing with a murine horseradish peroxidase (HRP) conjugated antibody (New England BioLabs) against MBP at 1:50,000 dilution. Bands were detected by chemiluminescence using enhanced chemiluminescent (ECL) substrate (Thermo Scientific) and a digital imaging system (ImageQuant^TM^ LAS 4000, GE Healthcare).

## Additional Information

**How to cite this article**: Pandhare, A. *et al*. Pentameric quaternary structure of the intracellular domain of serotonin type 3A receptors. *Sci. Rep.* **6**, 23921; doi: 10.1038/srep23921 (2016).

## Figures and Tables

**Table 1:** SEC-MALS analysis.

n	Molar mass (*M*_w_,10^3^ g/mole)	Dispersity (*Ð* =* M*_*w*_/*M*_*n*_)
1	248.6	1.018
2	257.2	1.015
3	258.5	1.009
4	239.2	1.001
